# Supplementary material for: Physiological demands and motion analysis of elite foil fencing
Source: PLoS One. 2023 Feb 14;18(2):e0281600. doi: 10.1371/journal.pone.0281600 (PMC9928104; doi:10.1371/journal.pone.0281600)
Supplement: Attachment — Submitted filename: Rebuttal Letter.docx [file pone.0281600.s001.docx]

Rebuttal Letter

Thank you for reviewing our paper. We have gone through the feedback and tried to make the amendments suggested. Hopefully this has improved the manuscript.

**Reviewer #1:** L43. Please provide a reference that fencing with foil, epee or sabre has different physiological demands and clarify that it is the fencing with different weapons that may induce different physiological demands.

**Response**: Thank you for your comment, we have now included a reference and added the word tactical to clarify why they are different.

**Reviewer**: L121. Please reconsider whether it was allocated and not randomized.

**Response**: We have deleted randomised and called it counterbalanced.

**Reviewer**: Ls 138-141. There is repeat here of info also communicated partly in Ls 118-123.

**Response**: Thank you, we have now deleted the repeated information.

**Reviewer**: L143. Please reconsider “The order of the fights were randomised and counterbalanced, to minimise the effect of fatigue”. It is not clear how starting with the most demanding fencing would minimize fatigue.

**Response**: Thank you for the comment, to avoid confusion we have deleted the sentence.

**Reviewer**: L154. I suggest to change BM·H-2 with “kg·m-2”.

**Response**: Thank you, we have now changed it to the recommended.

**Reviewer**: L171. The statement “The maximum value of the samples measured in minutes 1 and 3 was calculated, according to previous studies” is unclear why you need calculation. Please clarify.

**Response**: We have amended the wording to “the maximum value of the samples measured in minutes 1 and 3 was taken as maximum, according to previous studies”

**Reviewer**: L211. The dot on the first E in EE can be removed. Do this throughout the manuscript.

**Response**: We have now removed the dot throughout.

**Reviewer**: L255. Please check “0.06 mmol∙L-1 (95% CI, 0.26 to 0.38 mmol∙L-1)”. Is the 95% CI for the change of 0.06?

**Response**: Thanks for the query, I have gone and double checked the SPSS output and that is correct, although I missed the minus sign. The difference is 0.06 and it is the 95% CI for the difference in the pairwise comparisons. Hopefully it makes sense with the minus sign now. Apologies for missing it.

**Reviewer**: L265. Please provide the age-predicted equation in the methods.

**Response**: Thank you for spotting this, we have now included the equation in the methods.

Editor Comments:

1. Please ensure that your manuscript meets PLOS ONE's style requirements, including those for file naming. The PLOS ONE style templates can be found at
   https://journals.plos.org/plosone/s/file?id=wjVg/PLOSOne_formatting_sample_main_body.pdf and 
   <https://journals.plos.org/plosone/s/file?id=ba62/PLOSOne_formatting_sample_title_authors_affiliations.pdf>

**Response**: We have gone through and tried to ensure the manuscript meets PLOS ONE’s style requirements. Apologies for not having made sure it met the guidelines before.

2. We appreciate the funding received from the Ministerio de Universidades (Spain) for mobility stays for professors and researchers in foreign centers of higher education and research, the support of the Institut Nacional d’Educació Física de Catalunya (INEFC), Spanish Fencing Federation and CAR Sant Cugat.

We note that you have provided funding information that is not currently declared in your Funding Statement. However, funding information should not appear in the Acknowledgments section or other areas of your manuscript. We will only publish funding information present in the Funding Statement section of the online submission form. 
Please remove any funding-related text from the manuscript and let us know how you would like to update your Funding Statement. Currently, your Funding Statement reads as follows:

The authors received no specific funding for this work.

Please include your amended statements within your cover letter; we will change the online submission form on your behalf.

**Response**: We have deleted the comment about funding in the acknowledgements section. We have amended the cover letter to include a statement about funding. Apologies for this oversight.

3. We note that you have stated that you will provide repository information for your data at acceptance. Should your manuscript be accepted for publication, we will hold it until you provide the relevant accession numbers or DOIs necessary to access your data. If you wish to make changes to your Data Availability statement, please describe these changes in your cover letter and we will update your Data Availability statement to reflect the information you provide.

**Response**: I am just sorting out how to upload it to our University’s repository and I will then be able to provide a DOI.

4. Please review your reference list to ensure that it is complete and correct. If you have cited papers that have been retracted, please include the rationale for doing so in the manuscript text, or remove these references and replace them with relevant current references. Any changes to the reference list should be mentioned in the rebuttal letter that accompanies your revised manuscript. If you need to cite a retracted article, indicate the article’s retracted status in the References list and also include a citation and full reference for the retraction notice.

**Response**: The reference list should be complete.
